# A43 HIGH MUC2 MUCIN BIOSYNTHESIS BY GOBLET CELLS UNDER METABOLIC STRESS PRODUCE ELEVATED LEVELS OF PRO-INFLAMMATORY CYTOKINES

**DOI:** 10.1093/jcag/gwab049.042

**Published:** 2022-02-21

**Authors:** A Kim, F Moreau, H Gorman, K Chadee

**Affiliations:** University of Calgary Cumming School of Medicine, Calgary, AB, Canada

## Abstract

**Background:**

The colonic mucus bilayer is an integral innate host defense mechanism that houses the commensal gut microbiota whilst protecting the underlying mucosa against harmful pathogens. This essential barrier is generated via a highly ER stressful mechanism by goblet cells that produce MUC2 mucin. Goblet cells also produce a variety of proteins and peptides as well as pro-inflammatory cytokines that interact with and activate other arms of the innate immune system. In ulcerative and infectious colitis, goblet cells hyper secrete mucus and become depleted of their mucin stores. However, it is not known if other proteins or pro-inflammatory responses are altered in mucin-depleted goblet cells. Here, we elucidated the molecular mechanisms that regulate pro-inflammatory responses in WT and CRISPR/Cas9 MUC2 KO human goblet cells.

**Aims:**

Hypothesis: MUC2 biosynthesis and production augment pro-inflammatory responses in goblet cells. The specific aims are:

1. To determine the pro-inflammatory responses in WT and MUC2KO goblet cells

2. To determine if the MAPK signaling pathway is altered in WT and MUC2KO goblet cells

**Methods:**

mRNA and protein expression of various pro-inflammatory chemokines/cytokines in WT and CRISPR/Cas9 MUC2KO LS174T goblet cells were analyzed by RT-qPCR and 15-plex Luminex array. IL-22 was used to alleviate ER stress, as quantified by the expression of stress proteins by Western blotting. To determine differences in pro-inflammatory cytokine release, cells were stimulated with phorbol myristate acetate (PMA, positive control), live *Entamoeba histolytica (Eh),* and lysed soluble amebic proteins (SAP). MAPK signaling was enumerated by specific pharmacological inhibitors and analyzed by Western blotting.

**Results:**

Basally and in response to live *Eh*, WT goblet cells expressed high levels of the metabolic stress proteins, ATF4, GRP78, and CHOP as compared to MUC2KO cells. In response to PMA, live *Eh,* and SAP, WT cells expressed high levels of mRNA transcripts and secreted significant amounts of the pro-inflammatory chemokines, monocyte chemoattractant protein-1 (MCP-1), interleukin-8 (IL-8), and interleukin-13 (IL-13) as compared to MUC2KO cells. Reducing ER stress with IL-22 significantly augmented IL-8 protein release in both WT and MUC2KO cells. Signaling via the ERK MAPK pathway was elevated in WT whereas in MUC2KO goblet the response was delayed.

**Conclusions:**

This study demonstrates that high MUC2 mucin biosynthesis regulates the expression and secretion of the pro-inflammatory cytokines MCP-1, IL-8, and IL-13. Mechanistically, MUC2 induced metabolic stress regulated MAPK activity for high output pro-inflammatory responses as compared to MUC2KO goblet cells.

**Funding Agencies:**

CIHR

